# Signalling C‐Type lectin receptors, microbial recognition and immunity

**DOI:** 10.1111/cmi.12249

**Published:** 2014-01-10

**Authors:** J. Claire Hoving, Gillian J. Wilson, Gordon D. Brown

**Affiliations:** ^1^ Institute of Infectious Disease and Molecular Medicine Faculty of Health Sciences University of Cape Town Observatory Cape Town South Africa; ^2^ Aberdeen Fungal Group University of Aberdeen Institute of Medical Sciences Foresterhill Aberdeen UK

## Abstract

Signalling C‐type lectin receptors (CLRs) are crucial in shaping the immune response to fungal pathogens, but comparably little is known about the role of these receptors in bacterial, viral and parasitic infections. CLRs have many diverse functions depending on the signalling motifs in their cytoplasmic domains, and can induce endocytic, phagocytic, antimicrobial, pro‐inflammatory or anti‐inflammatory responses which are either protective or not during an infection. Understanding the role of CLRs in shaping anti‐microbial immunity offers great potential for the future development of therapeutics for disease intervention. In this review we will focus on the recognition of bacterial, viral and parasitic pathogens by CLRs, and how these receptors influence the outcome of infection. We will also provide a brief update on the role of CLRs in antifungal immunity.

## Introduction

The innate immune system provides the first line of defence against microbial attack, and is induced by recognition of microbial components, known as pathogen‐associated molecular patterns (PAMPs) or microbial‐associated molecular patterns (MAMPs), by pattern recognition receptors (PRRs). PAMPs are highly conserved and generally unique to microbes (Akira *et al*., 2006). Fungal PAMPs consist primarily of cell wall carbohydrate structures, while bacterial PAMPS range from lipoproteins, lipopolysaccharide (LPS), flagellin and peptidoglycan to bacterial nucleic acid structures. Viruses on the other hand are mainly recognized through their nucleic acids, such as double (dsRNA) or single stranded‐RNA (ssRNA) and viral DNA, although surface envelope glycoproteins can also be recognized. Although the PAMPs are not as well characterized, parasites, particularly helminths such as *Schistosoma mansoni* and *Trichuris muris*, or protozoa, such as *Leishmania infantum* and *Plasmodium berghei*, are also recognized by mammalian PRRs (McGuinness *et al*., 2003; Broz and Monack, 2013; Drummond and Brown, 2013).

**Figure 1:**
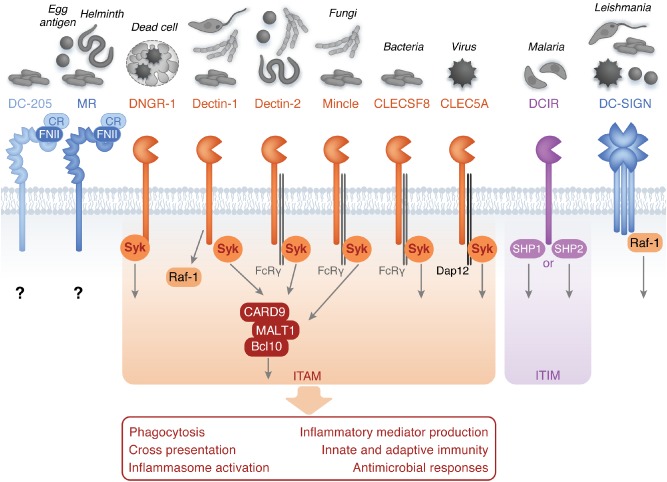
Recognition of microorganisms by signalling CLRs. Cartoon representation of the C‐type lectin receptors discussed in the text. Also shown is the microbes they recognize, the major intracellular signalling pathways utilized by these receptors, and the responses they induce. ITAM indicates receptors utilizing immunoreceptor tyrosine‐based activation motifs; ITIM indicates receptors utilizing immunoreceptor tyrosine‐based inhibitory motifs. CR (cysteine‐rich domain), FNII (fibronectin domain).

PRR recognition of a PAMP can lead to the activation of intracellular signalling pathways that elicit innate responses against pathogens and direct the development of adaptive immunity. Also important to mention is the recognition of damage‐associated molecular patterns (DAMPs) by PRRs. Molecules released by stressed cells or cells undergoing necrosis can act as danger signals and promote inflammatory responses (see for example Yamasaki *et al*., 2008). PRRs of relevance here are the signalling trans‐membrane C‐type lectin receptors (CLRs), which are widely recognized to play an essential role in antifungal immunity (see Hardison and Brown, 2012 for a recent review). Less well recognized is their role in immunity to other microorganisms. This review will therefore focus on the role of signalling CLRs in immunity to bacteria, viruses, helminths and protozoa, and will only briefly discuss the most recent advances in our understanding of their role in antifungal immunity. Furthermore, we have included certain important CLRs in which the signalling pathway is unclear, particularly those from Group VI. The structures of selected CLRs that will be discussed are represented in Fig. 1.

## C‐type lectin receptors: general overview

CLRs comprise a large family of receptors which bind carbohydrates, through one or more carbohydrate recognition domains (CRDs), or which possess structurally similar C‐type lectin‐like domains (CTLDs) which do not necessarily recognize carbohydrate ligands. CLRs are divided into 17 groups based on features including phylogeny and structure (Zelensky and Gready, 2005). Discussed in this review are CLRs from groups II (calcium‐dependent lectins with single CRDs), group V (calcium‐independent receptors with single CTLDs) and group VI (calcium‐dependent lectins with multiple CRDs) (see Table 1). Based on their signalling potential, CLRs can be further subdivided into (i) activation Syk‐coupled CLRs with immunoreceptor tyrosine‐based activation motif (ITAM) domains, (ii) inhibitory CLRs with immunoreceptor tyrosine‐based inhibition motif (ITIM) domains or (iii) CLRs without clear ITAM or ITIM domains such as MR, DEC‐205 and DC‐SIGN (Zelensky and Gready, 2005; Sancho and Reis e Sousa, 2012).

**Table 1:** Selected CLRs mentioned in this review

	CLR	Ligands	Ligand origin	Selected references
Group II: Calcium‐dependent CRD	Dectin‐2	α‐mannans O‐linked mannobiose‐rich glycoprotein	*M. tuberculosis* *S. mansoni* SEA *C. albicans* *Malassezia* spp. HDM allergens	Ritter *et al*., 2010; Meevissen *et al*., 2012; Ishikawa *et al*., 2013; Prasanphanich *et al*., 2013; Salazar *et al*., 2013
	CLECSF8	TDM	*M. bovis* *K. pneumonia*	Miyake *et al*., 2013; Steichen *et al*., 2013
	Mincle	α‐mannose mannitol‐linked glyceroglycolipid mannosyl fatty acids TDM	*M. tuberculosis* *C. albicans* *Malassezia* spp.	Ishikawa *et al*., 2009; Lee *et al*., 2012; Sancho and Reis e Sousa, 2012; Ishikawa *et al*., 2013
	DC‐SIGN	High mannose SlpA	HIV‐1 Measles Dengue *Mycobacterium* spp. *Influenza A* SEA *Leishmania* spp. *Helicobacter pylori* *Lactobacillus* spp.	Gringhuis *et al*., 2007; Konstantinov *et al*., 2008; Geijtenbeek and Gringhuis, 2009; Mesman *et al*., 2012; Avota *et al*., 2013; Chen *et al*., 2013; Harman *et al*., 2013; Hillaire *et al*., 2013
	SIGNR3	High mannose and fucose	*L. infantum* SEA	Powlesland *et al*., 2006; Meevissen *et al*., 2012; Lefevre *et al*., 2013; Prasanphanich *et al*., 2013
	SIGNR1	High mannose and fucose	SEA	Galustian *et al*., 2004; Meevissen *et al*., 2012; Prasanphanich *et al*., 2013
	MGL	Lewis X	SEA	Van Vliet *et al*., 2005; Meevissen *et al*., 2012; Tundup *et al*., 2012
	DCIR	unknown	HIV‐1	Sancho and Reis e Sousa, 2012
				
Group V: Calcium‐independent non‐CRD	Dectin‐1	β‐glucans	*L. infantum* *C. albicans* *Mycobacterium* spp.	Hardison and Brown, 2012; Lefevre *et al*., 2013
	CLEC5A	Unknown	Dengue virus JEV	Chen *et al*., 2012; Drummond *et al*., 2013
	DNGR‐1 (CLEC9A)	F‐actin	Vaccinia virus Herpes simplex virus	Iborra *et al*., 2012; Zelenay *et al*., 2012
				
Group VI: Calcium‐dependent multiple CRD	Mannose Receptor (MR)	High mannose Omega‐1 ManLam	SEA *Trichuris muris* *Mesocestoides corti* HDM allergens *Mycobacterium* spp. *K. pneumonia* *S. pneumonia* *F. tularesis*	Kang *et al*., 2005; Deschoolmeester *et al*., 2009; Geijtenbeek and Gringhuis, 2009; Everts *et al*., 2012; Meevissen *et al*., 2012; Mishra *et al*., 2013; Prasanphanich *et al*., 2013; Salazar *et al*., 2013
	DEC‐205 (CD205)	PLA	*Y. pestis*	Zhang *et al*., 2008

Relevant references are indicated in text.

HDM, house dust mite; HIV, human immunodeficiency virus; JEV, Japanese encephalitis virus ManLam, mannosylated lipoarabinomannan; PLA, plasminogen activator; SEA, schistosoma egg antigen; SlpA, surface layer A protein; TDM, trehalose‐6,6′‐dimycolate.

Activation of CLRs can induce intracellular signalling pathways in two ways: firstly through indirect signalling, where receptors such as macrophage‐inducible C‐type lectin (Mincle or CLEC4E), Dectin‐2 (CLEC6A) and C‐type lectin domain family 5A (CLEC5A) associate with ITAM containing adaptor molecules such as Fc Receptor γ‐chain (FcRγ) or DAP12.

The second mechanism employed by Dectin‐1 (or CLEC7A) and DNGR‐1 (CLEC9A) involves direct signalling through ITAM‐like motifs located within the cytoplasmic tail of these receptors (Rogers *et al*., 2005; Geijtenbeek and Gringhuis, 2009). Signalling via both mechanisms involves the recruitment to phosphorylated tyrosine residues of spleen tyrosine kinase (Syk), which in turn co‐ordinates a complex of CARD9, B cell lymphoma 10 (Bcl10) and mucosa‐associated lymphoid tissue lymphoma translocation protein 1 (Malt1). Furthermore, apoptosis‐associated speck‐like protein containing a CARD (ASC) has also been shown to be phosphorylated in a Syk‐ and Jnk‐dependent manner (Hara *et al*., 2013). Protein kinase C‐δ (PKCδ) is also an essential element in this pathway (Strasser *et al*., 2012). Signalling from Dectin‐1 and other lectins also involves additional pathways, such as the Syk‐independent activation of Raf‐1 (Gringhuis *et al*., 2009). These signalling pathways lead to activation of several downstream molecules, including NFκβ and mitogen‐activated protein kinases (MAPK), eventually triggering cellular responses, including phagocytosis, DC maturation, chemotaxis, the respiratory burst, inflammasome activation, and cytokine and other mediator production (Drummond *et al*., 2011; Strasser *et al*., 2012). Moreover, these receptors can also drive the development of adaptive immunity, particularly T helper (Th)1 and Th17 CD4^+^ and CD8^+^ T cell adaptive responses, although some CLRs are also capable of driving Th2 immunity (Kerrigan and Brown, 2011; Sancho and Reis e Sousa, 2012).

While signalling through ITAM‐bearing receptors results in cell activation, ITIM‐bearing receptors usually mediate inhibitory functions. For example, DCIR contains an ITIM in its cytoplasmic tail which recruits tyrosine phosphatases (SHP‐1 and SHP‐2) following ligand binding to modulate the signalling pathways induced by other PRRs, such as inhibition of TLR8‐ or TLR9‐induced cytokine production (Geijtenbeek and Gringhuis, 2009). However, the function of inhibitory CLRs is not always clear cut as these receptors can also have activation functions, thereby mediating cellular activation. They may either recruit novel substrates to their cytoplasmic domains or inhibit other inhibitory receptors (Redelinghuys and Brown, 2011).

## The role of C‐type lectins in microbial recognition and immunity

### Bacteria

The role of CLRs in anti‐bacterial immunity has been best characterized for *M. tuberculosis* (MTB). Multiple CLRs including Dectin‐1, DC‐SIGN, mannose receptor (MR), and Mincle have been implicated in MTB control *in vitro*, however each of these CLRs appears to be redundant in controlling infection *in vivo*. For example, Dectin‐1 was shown to be required for the production of IL‐12p40 by splenic DCs in response to mycobacteria *in vitro*, but was redundant during infection *in vivo* (Rothfuchs *et al*., 2007; Court *et al*., 2010; Marakalala *et al*., 2010). MR binds to mannose‐capped lipoarabinomannan (ManLAM) on the surface of *M. tuberculosis*, mediating bacterial phagocytosis and limiting phagosome–lysosome fusion within macrophages, but loss of the MR did not influence antimycobacterial immunity *in vivo* (Schlesinger *et al*., 1994; Kang *et al*., 2005; Court *et al*., 2010). Mincle mediates recognition of mycobacterial cord factor, trehalose‐6,6′‐dimycolate (TDM), and was shown to be essential for driving immune responses to TDM *in vivo*, including granuloma formation (Ishikawa *et al*., 2009). Yet Mincle knockout mice had normal granulomas and did not show obvious defects during live MTB infection (Lee *et al*., 2012; Heitmann *et al*., 2013). DC‐SIGN recognizes ManLAM and α‐glucan, preventing DC maturation and the production of IL‐10 (Geijtenbeek *et al*., 2003; Geurtsen *et al*., 2009). Although mice deficient in a murine homologue for DC‐SIGN, SIGNR3^−/−^ showed defects in early control of MTB, they mounted an efficient anti‐mycobacterial adaptive immune response with granulomatous lesions comparable to wild‐type controls (Tanne *et al*., 2009).

Despite the apparent redundancy of these receptors *in vivo*, the shared CLR downstream signalling pathway involving CARD9 is critical for protection, as CARD9^−/−^ mice present with uncontrolled bacterial replication and exacerbated neutrophilic pulmonary inflammation, which is followed by death (Dorhoi *et al*., 2010). This suggests either that a combination of CLRs or an unknown CLR is essential for protection against MTB. Recently studies have shown that Mincle can form a receptor complex with CLECSF8 (CLEC4D or MCL) and FcεRIγ, and this heterotrimeric complex is proposed to be the functionally optimal form for these CLRs (Lobato‐Pascual *et al*., 2013). However, the role of CLECSF8 in live MTB infection has not been defined.

Recently, CLECSF8 was also shown to be important in the resolution of pneumonia caused by *Klebsiella pneumoniae* (Steichen *et al*., 2013). CLECSF8^−/−^ mice were more susceptible than wild‐type mice to pneumonic sepsis, with increased bacterial burdens, hyper‐inflammation and severe lung pathology which correlated with a massive accumulation of neutrophils. These results suggest that CLECSF8 plays an important role in resolution of inflammation, and is the first report describing a physiological function for this CLR.

In addition to *M. tuberculosis*, DC‐SIGN also interacts with a wide range of other bacterial pathogens including *M. leprae*, *Helicobacter pylori* and Lactobacillus species (Geijtenbeek and Gringhuis, 2009). *Lactobacillus reuteri* and *L. casei* have been shown to bind DC‐SIGN and induce regulatory T‐cells (Smits *et al*., 2005), while surface (S) layer A protein (SlpA) on the surface of *L. acidophilus* has been identified as a ligand of this CLR (Konstantinov *et al*., 2008). The MR also recognizes a number of other mannose‐expressing bacterial species, including *M. kansasii*, *K. pneumonia*, *Streptococcus pneumoniae* and *Francisella tularensis*. However, the MR seems not to be essential during infection with these pathogens *in vivo* (Geijtenbeek and Gringhuis, 2009).

Other less well characterized CLRs have also been shown to recognize bacteria. DEC‐205, for example, is a member of the mannose receptor family and binds to plasminogen activator (PLA) on the surface of *Yersinia pestis*, which mediates bacterial attachment (Lähteenmäki *et al*., 1998). This CLR was found to promote dissemination of this pathogen, and this had detrimental implications for the host (Zhang *et al*., 2008).

### Viruses

Viruses are abundant, rapidly evolving pathogens which pose a continual challenge to the host immune system. Unlike the predominantly protective responses that CLRs mediate to other pathogens, viral recognition by CLRs tends to favour transmission, infection and inflammation. Recognition of HIV by DC‐SIGN is a well characterized example of the detrimental effect of CLR signalling in response to a virus. Firstly, binding of HIV‐1 to DC‐SIGN not only modulates TLR‐induced IL‐10 production by signalling via Raf‐1, but also impairs T cell proliferation and TLR‐induced dendrite formation of DCs (Gringhuis *et al*., 2007). Secondly, HIV gp120 facilitates DC‐SIGN‐mediated viral entry into the cells, results in infected CD4^+^ target cells (Harman *et al*., 2013) and accelerated DC apoptosis, negatively affecting DC maturation which would normally promote pathogen recognition by the immune system (Chen *et al*., 2013). Therefore, preventing HIV from binding to mucosal DCs by blocking CLRs could potentially prevent HIV transmission.

DC‐SIGN ligands are not limited to HIV but include a range of other viruses, such as Cytomegalovirus, Dengue virus, Ebola virus, Hepatitis C virus, SARS‐coronavirus, West Nile virus and the Measles virus (Mesman *et al*., 2012; Avota *et al*., 2013; Hillaire *et al*., 2013). More recently, DC‐SIGN was shown to assist in the replication of Influenza A virus, by binding to glycans on haemagglutinin, promoting viral binding to cells and internalization *in vitro* (Hillaire *et al*., 2013). However, the contribution of DC‐SIGN was dependent on the extent of the glycosylation of viral haemagglutinin.

Viral exploitation of CLRs can also induce pro‐inflammatory cytokines leading to severe pathology for the host. Previously, MR and CLEC5A have been shown to bind Dengue virus *in vitro* leading to infection of macrophages and inflammasome activation respectively. Unlike conventional CLRs which are involved in Dengue virus entry into target cells, CLEC5A regulates virus‐induced pro‐inflammatory cytokines and blocking CLEC5A‐mediated signalling attenuates pro‐inflammatory cytokine production by infected macrophages, reducing mortality, and maintaining host immunity, leading to resolution of infection. This indicates that CLEC5A is critical in regulating inflammatory reactions triggered by pathogens. The elevated levels of TNF‐α during infection were associated with DAP12 activation, suggesting that CLEC5A directly interacts with the Dengue virion (Chen *et al*., 2008). Similarly, Japanese encephalitis virus (JEV) also binds CLEC5A directly, and induces neuro‐inflammation through DAP12 activation in macrophages. Blocking CLEC5A reduced neuronal damage, pro‐inflammatory cytokine secretion, blood‐brain barrier permeability, and cellular infiltration into the central nervous system (CNS) *in vivo* (Chen *et al*., 2012). Together these studies suggest that CLR blockage could alleviate tissue damage and increase survival of patients with virus‐induced inflammatory diseases.

In contrast to these detrimental roles, CLRs can also induce protective responses. This is exemplified by DNGR‐1 (CLEC9A) in the control of both vaccinia (Iborra *et al*., 2012) and Herpes Simplex (Zelenay *et al*., 2012) viruses. DNGR‐1 is expressed by a subset of DC's and detects dead cells, promoting antigen cross‐presentation to CD8^+^ T cells. Although DNGR‐1‐deficient DCs are activated following interaction with virus‐infected cells, they are no longer capable of cross‐presenting antigens. This results in weaker CD8^+^ T‐cell responses, delayed lesion resolution and a higher viral load, suggesting that tissue damage sensing by DNGR‐1 is a key component in anti‐viral immunity.

Another example of protective responses mediated by CLRs is the role of DCIR during infection with Chikungunya virus (Long *et al*., 2013). DCIR^−/−^ mice developed more severe inflammatory disease with a skewed cytokine response both *in vivo* and *in vitro*. Thus the inhibitory functions of this receptor play an important role in suppressing pathological inflammatory responses induced by this pathogen (Long *et al*., 2013).

### Helminths

Helminth parasites drive host CD4^+^ Th cells toward Th2 and anti‐inflammatory responses, and induce alternative activation of macrophages. Helminth glycans are thought to play a critical role in driving these responses, and given the large number of glycan moieties they possess; it is likely that many CLRs are involved in immunity to these pathogens. Soluble egg antigen (SEA) of *Schistosoma mansoni* cercariae was the first parasite‐specific ligand for DC‐SIGN described (Meyer *et al*., 2005). Subsequently, glycoproteins from SEAs of several schistosome species (*S. mansoni*, *S. hematobium*, *S. japonicum*) have been described as ligands for DC‐SIGN (van Die *et al*., 2003).

Other signalling CLRs have been shown to recognize SEA or *S. mansoni* glycans, although not all of the actual ligands have been identified. Among these, Dectin‐2 recognizes an unknown ligand in SEA; SIGNR1, SIGNR3 and MR all recognize Lewis *x* and high‐mannose N‐glycans; and macrophage galactose lectin (MGL) recognizes Lewis x, LDN and LDN‐F in SEA (Meevissen *et al*., 2012; Prasanphanich *et al*., 2013). Furthermore, MR recognizes Omega‐1 in SEA and conditions DCs for Th2 priming (Everts *et al*., 2012). Dectin‐2, in particular, was the first Syk‐coupled CLR to be associated with helminth infections, and plays a role in regulating helminth immune responses by indirectly reducing Th2‐mediated pathology. Here, Dectin‐2 was found to induce active IL‐1β secretion by activating the Nlrp3 inflammasome in response to *S. mansoni* SEA (Ritter *et al*., 2010).

Other CLRs have also been implicated in anti‐helminth immunity but their role *in vivo* is less clear. For example, murine SIGNR1 (a homologue of DC‐SIGN) binds SEA *in vitro*, but SIGNR1^−/−^ mice mount a normal response during *S. mansoni* infection. Both MGL and DC‐SIGN are able to recognize SEA antigens, but the importance of these interactions *in vivo* is still unknown (Meevissen *et al*., 2012; Tundup *et al*., 2012). Similarly, MR was shown to bind *Trichuris muris* excretory/secretory proteins but did not affect parasite clearance, as MR^−/−^ mice cleared the infection normally (Deschoolmeester *et al*., 2009).

Notably, a recent publication has shown a role for MR against *Mesocestoides corti*, a tape worm that releases glycan antigens within the CNS causing neurocysticercosis (Mishra *et al*., 2013). *In vivo*, MR^−/−^ mice have increased survival, with accumulation of regulatory granulocytic myeloid cells and reduced T cell numbers. Therefore, the pathogenesis of neurocysticercosis appears to be directly attributable to the immune response against the parasite induced by MR. Although these *in vivo* results should be interpreted with caution due to the fact that a microRNA (miR‐511‐1) is encoded within the MR gene and co‐regulated with MR, possibly influencing the observed phenotype (Tserel *et al*., 2011).

In the context of Th2 immunity, CLRs also play a key role in promoting allergic responses. Of particular importance are MR, DC‐SIGN and Dectin‐2 (Salazar *et al*., 2013). While, MR and DC‐SIGN both recognize *Dermatophagoides pteronyssinus* group 1 antigen (Der p 1) from house dust mite (HDM), Dectin‐2 recognizes both HDM (*D. farinae* and *D. pteronyssinus*) and mold (*Aspergillus fumigatus*) extracts. Dectin‐2 was found to trigger the generation of cysteinyl leucotrienes (cys‐LT), which mediated pulmonary inflammation. In absence of LTC_4_ synthase (a critical enzyme in cys‐LT generation) or the cys‐LT receptor, pulmonary inflammation was reduced (Barret *et al*., 2009).

### Protozoa

Several CLRs have been implicated in the recognition of *Leishmania* species, but their role is only starting to be understood. DC‐SIGN and L‐SIGN (a close homologue of DC‐SIGN which also recognizes high‐mannose glycans) molecule have both been shown to recognize *Leishmania*, but the receptors differ in their ability to interact with these organisms, depending on the species and the stage of parasite maturation (Caparros *et al*., 2005). Dectin‐1 and MR have been shown to be crucial for the ‘killing’ response against *L. infantum*, by inducing ROS in macrophages and *triggering Syk‐coupled secretion of IL‐1β* (Lefevre *et al*., 2013). SIGNR3, on the other hand, has been lined with parasite survival both *in vivo* and *in vitro*, by inhibiting the LTB4/IL‐1β axis (Lefevre *et al*., 2013). As LTB4 is known to play a crucial role in the mechanisms responsible for killing *Leishmania*, specifically through the activation of IL‐1β; it has been suggested that elevated LTB4 benefits the host while reduced LTB4 benefits the pathogen. These studies highlight the divergent but essential roles of CLRs in *Leishmania* pathogenesis.

CARD9 has recently been linked to cerebral malaria (CM), where its expression was upregulated in a mouse model induced by Plasmodium Berghei (Hafalla *et al*., 2012). However, CARD9^−/−^ mice were not protected from infection, suggesting that CM develops independently of CARD9 despite its upregulation during disease. In contrast, striking protection against CM was observed when DNGR‐1^+^ DCs were depleted. Protection was associated with reduced numbers of CD8^+^ cells, reduced parasite burdens in the brain and reduced IFN‐γ levels (Piva *et al*., 2012). More recently a study using DCIR^−/−^ mice also demonstrated significant survival compared with wild‐type controls. Protection was associated with reduced CD8^+^ cells and reduced brain inflammation, highlighting the activation functions of some inhibitory receptors (Redelinghuys and Brown, 2011; Maglinao *et al*., 2013). Although the specific mechanism underlying this protection is unknown, it is clear that CLRs play a critical role in CM.

### Fungi

Innate and adaptive immune responses to fungi are primarily mediated by CLRs, with Dectin‐1 being the best characterized in the context of fungal infections (Hardison and Brown, 2012). For fungal infections innate and adaptive immune responses are primarily regulated by CLRs. Due to the rapid increase of advances made in this field we will discuss some of the recent studies which have dissect CLR‐mediated mechanisms involved in antifungal immunity. Dectin‐1 recognizes β‐glucan and induces multiple cellular functions through its cytoplasmic signalling domain, and is essential for protective immune response to *Candida albicans* and other fungi in mice and humans. Recently we have discovered that the requirement for Dectin‐1 in the control of *C. albicans* is strain specific, as different *C. albicans* strains have variations in the composition and nature of their cell walls which only become apparent during infection *in vivo* (Marakalala *et al*., 2013). Dectin‐1‐mediated protection to *C. albicans* infections has also recently been attributed to the production of type I IFN by renal infiltrating DCs, a response which required Syk, CARD9 and IRF5 (del Fresno *et al*., 2013). Other signalling CLRs which play important roles include Dectin‐2, Mincle, DC‐SIGN and the MR (Hardison and Brown, 2012).

The influence of the fungal microbiota on immune regulation is another recent area of progress. The mammalian intestinal microbiota was found to include a myriad of fungal species, with over 100 known and 100 novel fungal species being identified (Iliev *et al*., 2012). Mice lacking Dectin‐1 were shown to have increased susceptibility to dextran sodium sulfate (DSS) induced colitis when specific fungal species were present in their gastrointestinal tract. Moreover, a polymorphism of Dectin‐1 was identified which associated with patients presenting with severe ulcerative colitis (Iliev *et al*., 2012). Similarly, SIGNR3 recognition of fungi was shown to influence immune regulation in the gut, as SIGNR3^−/−^ mice exhibit an exacerbated DSS‐induced colitis compared with wild‐type controls (Eriksson *et al*., 2013). Together, these studies highlight the importance of fungal recognition by CLRs and the role this plays in maintaining intestinal immune homeostasis and control of disease.

As we have already discussed, CLRs are important in initiating innate immunity and link pathogen recognition to the development of adaptive immunity. More recently, the concept of innate immune memory or ‘trained immunity’ has emerged and challenged conventional paradigms of T and B cell‐mediated adaptive memory. Essentially, trained immunity is induced after a primary infection or vaccination, confers protection independently of T or B cells, mediated by innate immune cells such as NK cells and monocytes/macrophages, and increases resistance to infection by the same or other pathogens (Netea *et al*., 2011; Netea, 2013). Trained immunity can be distinguished from immune priming due to the fact that after recovery from infection, innate immune responses do not return to the steady‐state level. This is due to the epigenetic reprogramming of innate immune cells rather than the short‐lived change of state seen in immune priming. Dissecting the mechanisms involved in trained immunity provides an exciting new approach to protection against infection. A role for CLRs in trained immunity has recently been described, where the reprogramming of monocytes by Dectin‐1/ Raf‐1 signalling prevented infection with *C. albicans* and other organisms (Quintin *et al*., 2012). Insights into trained immunity triggered by CLRs could therefore form the basis for novel strategies in immunotherapy and vaccination.

Lastly, it is well established that collaboration between CLRs and TLRs initiates optimal antifungal responses. In fact, the collaborative responses induced by Dectin‐1 and TLR2 was one of the first collaborative PRR responses ever described (Hardison and Brown, 2012). A more recent example of the importance of such collaboration is the recognition of *Fonsecaea pedrosoi*. This organism, which causes chromoblastomycoses, was shown to be recognized by CLRs, but not TLRs, and this resulted in defective inflammatory responses and susceptibility to infection. Amazingly, exogenous administration of TLR agonists restored protective inflammatory responses and led to clearance of the infection *in vivo* (Sousa *et al*., 2011). This approach is now being tested in humans (G.D.B., unpubl. data).

## Conclusions

It is well established that CLRs play an important role in recognizing fungi and orchestrate both innate and adaptive immune responses to these pathogens. Recent discoveries have revealed an ever increasing repertoire of pathogens that are also recognized by these receptors, including bacteria, helminths and protozoa. Moreover, we are discovering that CLRs play key roles in autoimmunity, allergy and in maintaining homeostasis. Yet we are only just beginning to understanding the importance of these receptors, and the next few years are likely to yield a wealth of exciting new breakthroughs.

## References

[cmi12249-bib-0001] Akira, S. , Uematsu, S. , and Takeuchi, O. (2006) Pathogen recognition and innate immunity. Cell 124: 783–801. 16497588 10.1016/j.cell.2006.02.015

[cmi12249-bib-0002] Avota, E. , Koethe, S. , and Schneider‐Schaulies, S. (2013) Membrane dynamics and interactions in measles virus dendritic cell infections. Cell Microbiol 15: 161–169. 22963539 10.1111/cmi.12025

[cmi12249-bib-0003] Barret, N.A. , Maekawa, A. , Rahman, O.M. , Austen, F. , and Kanaoka, Y. (2009) Dectin‐2 recognition of house dust mite triggers cysteinyl leukotriene generation by dendritic cells. J Immunol 182: 1119–1128. 19124755 10.4049/jimmunol.182.2.1119 PMC3682801

[cmi12249-bib-0004] Broz, P. , and Monack, D.M. (2013) Newly described pattern recognition receptors team up against intracellular pathogens. Nat Rev Immunol 13: 551–565. 23846113 10.1038/nri3479

[cmi12249-bib-0005] Caparros, E. , Serrano, D. , Puig‐Kroger, A. , Riol, L. , Lasala, F. , Martinez, I. , *et al*. (2005) Role of the C‐type lectins DC‐SIGN and L‐SIGN in Leishmania interaction with host phagocytes. Immunobiology 210: 185–193. 16164025 10.1016/j.imbio.2005.05.013 PMC7114652

[cmi12249-bib-0006] Chen, S. , Liu, R. , Wu, M. , Lin, Y. , Chen, S. , Tan, D.T. , *et al*. (2012) CLEC5A regulates Japanese encephalitis virus‐induced neuroinflammation and lethality. PLoS Pathog 8: e1002655. 22536153 10.1371/journal.ppat.1002655 PMC3334897

[cmi12249-bib-0007] Chen, S.T. , Lin, Y.L. , Huang, M.T. , Wu, M.F. , Cheng, S.C. , *et al*. (2008) CLEC5A is critical for dengue‐virus‐induced lethal disease. Nature 453: 672–676. 18496526 10.1038/nature07013

[cmi12249-bib-0008] Chen, Y. , Hwang, S. , Chan, V.S.F. , Chung, N.P.Y. , Wang, S. , Li, Z. , *et al*. (2013) Binding of HIV‐1 gp120 to DC‐SIGN promotes ASK‐1‐dependent activation‐induced apoptosis of human dendritic cells. PLoS Pathog 9: e1003100. 23382671 10.1371/journal.ppat.1003100 PMC3561151

[cmi12249-bib-0009] Court, N. , Vasseur, V. , Vacher, R. , Frémond, C. , Shebzukhov, Y. , *et al*. (2010) Partial redundancy of the PRRs, scavenger receptors, and C‐type lectins for the long‐term control of MTB infection. J Immunol 15: 7057–7070. 10.4049/jimmunol.1000164 20488784

[cmi12249-bib-0011] Deschoolmeester, M.L. , Martinez‐Pomares, L. , Gordon, S. , and Else, K.J. (2009) The mannose receptor binds Trichuris muris excretory/secretory proteins but is not essential for protective immunity. Immunology 126: 246–255. 18624733 10.1111/j.1365-2567.2008.02893.x PMC2632686

[cmi12249-bib-0012] van Die, I. , van Vliet, S.J. , Nyame, A.K. , Cummings, R.D. , Bank, C.M. , *et al*. (2003) The dendritic cell‐specific C‐type lectin DC‐SIGN is a receptor for *Schistosoma mansoni* egg antigen and recognizes the glycan antigen Lewis x. Glycobiology 13: 471–478. 12626400 10.1093/glycob/cwg052

[cmi12249-bib-0013] Dorhoi, A. , Desel, C. , Yeremeev, V. , Pradl, L. , Brinkmann, V. , Mollenkopf, H.J. , *et al*. (2010) The adaptor molecule CARD9 is essential for tuberculosis control. J Exp Med 12: 777–792. 10.1084/jem.20090067 PMC2856020 20351059

[cmi12249-bib-0014] Drummond, R.A. , and Brown, G.D. (2013) Signaling C‐type lectins in antimicrobial immunity. PLoS Pathog 9: e1033417. 10.1371/journal.ppat.1003417 PMC3723563 23935480

[cmi12249-bib-0015] Drummond, R.A. , Saijo, S. , Iwakura, Y. , and Brown, G.D. (2011) The role of Syk/CARD9 coupled C‐type lectins in antifungal immunity. Eur J Immunol 41: 276–281. 21267996 10.1002/eji.201041252 PMC3434674

[cmi12249-bib-0016] Eriksson, M. , Johannssen, T. , von Smolinski, D. , Gruber, A.D. , Seeberger, P.H. , and Lepenies, B. (2013) The c‐type lectin receptor SIGNR3 binds to fungi present in commensal microbiota and influences immune regulation in experimental colitis. Front Immunol 4: 196. 23882266 10.3389/fimmu.2013.00196 PMC3712271

[cmi12249-bib-0017] Everts, B. , Hussaarts, L. , Driessen, N.N. , Meevissen, M.H.J. , Schramm, G. , van der Ham, A.J. , *et al*. (2012) Schistosome‐derived omega‐1 drives Th2 polarization by suppressing protein synthesis following internalization by the mannose receptor. J Exp Med 209: 1753–1767. 22966004 10.1084/jem.20111381 PMC3457738

[cmi12249-bib-0018] del Fresno, C. , Soulat, D. , Roth, S. , Blazek, K. , Udalova, I. , Sancho, D. , *et al*. (2013) Interferon‐β production via dectin‐1‐Syk‐IRF5 signalling in dendritic cells in crucial for immunity to *C. albicans* . Immunity 38: 1176–1186. 23770228 10.1016/j.immuni.2013.05.010

[cmi12249-bib-0019] Galustian, C. , Park, C.G. , Chai, W. , Kiso, M. , Bruening, S.A. , Kang, Y.S. , *et al*. (2004) High and low affinity carbohydrate ligands revealed for murine SIGN‐R1 by carbohydrate array and cell binding approaches, and differing specificities for SIGN‐R3 and langerin. Int Immunol 16: 853–866. 15136555 10.1093/intimm/dxh089

[cmi12249-bib-0020] Geijtenbeek, T.B.H. , and Gringhuis, S.I. (2009) Signaling through C‐type lectin receptors: shaping immune responses. Nat Rev Immunol 9: 465–479. 19521399 10.1038/nri2569 PMC7097056

[cmi12249-bib-0021] Geijtenbeek, T.B.H. , Van Vliet, S.J. , Koppel, E.A. , Sanchez‐Herandez, M. , Vandenbroucke‐Grauls, C.M. , Appelmelk, B. , and van Kooyk, Y. (2003) Mycobacteria target DC‐SIGN to suppress dendritic cell function. J Exp Med 197: 7–17. 12515809 10.1084/jem.20021229 PMC2193797

[cmi12249-bib-0022] Geurtsen, J. , Chedammi, S. , Mesters, J. , Cot, M. , Driessen, N.N. , *et al*. (2009) Identification of mycobacterial alpha‐glucan as a novel ligand for DC‐SIGN: involvement of mycobacterial capsular polysaccharides in host immune modulation. J Immunol 183: 5221–5231. 19783687 10.4049/jimmunol.0900768

[cmi12249-bib-0023] Gringhuis, S.I. , den Dunnen, J. , Litjens, M. , van het Hof, B. , van Kooyk, Y. , and Geijtenbeek, T.B.H. (2007) C‐type lectin DC‐SIGN modulates toll‐like receptor signalling via Raf‐1 kinase‐dependent acetylation of transcription factor NF‐kappa B. Immunity 26: 605–616. 17462920 10.1016/j.immuni.2007.03.012

[cmi12249-bib-0024] Gringhuis, S.I. , den Dunnen, J. , Litjens, M. , van der Vlist, M. , Wevers, B. , Bruijns, J S.C. , and Geijtenbeek, T.B.H. (2009) Dectin‐1 directs T helper cell differentiation by controlling noncanonical NK‐kappaB activation through Raf‐1 and Syk. Nat Immunol 10: 203–213. 19122653 10.1038/ni.1692

[cmi12249-bib-0025] Hafalla, J.C. , Burgold, J. , Dorhoi, A. , Gross, O. , Ruland, J. , Kaufmann, S.H.E. , and Matuschchewski, K. (2012) Experimental cerebral malaria develops independently of CARD‐signalling. Infect Immun 80: 1274–1279. 22158744 10.1128/IAI.06033-11 PMC3294660

[cmi12249-bib-0026] Hara, H. , Tsuchiya, K. , Kawamura, I. , Fang, R. , Hernandez‐Cuellar, E. , Shen, Y. , *et al*. (2013) Phosphorylation of the adaptor ASC acts as a molecular switch that controls the formation of speck‐like aggregates and inflammasome activity. Nat Immunol 14: 1247–1255. 24185614 10.1038/ni.2749 PMC4813763

[cmi12249-bib-0027] Hardison, S.E. , and Brown, G.D. (2012) C‐type lectin receptors orchestrate anti‐fungal immunity. Nat Immunol 13: 817–822. 22910394 10.1038/ni.2369 PMC3432564

[cmi12249-bib-0028] Harman, A.N. , Kim, M. , Nasr, N. , Sandgren, K.J. , and Cameron, P.U. (2013) Tissue dendritic cells as portals for HIV entry. Rev Med Virol 23: 319–333. 23908074 10.1002/rmv.1753

[cmi12249-bib-0029] Heitmann, L. , Schoenen, H. , Ehlers, S. , Lang, R. , and Holscher, C. (2013) Mincle is not essential for controlling M. tuberculosis infection. Immunobiology 218: 506–516. 22784441 10.1016/j.imbio.2012.06.005

[cmi12249-bib-0030] Hillaire, L.B. , Nieuwekoop, N.J. , Boon, A.C.M. , de Mutsert, G. , Vogelzang‐van Trierum, S.E. , *et al*. (2013) Binding of DC‐SIGN to the Hemagglutinin of Influenza A viruses supports virus replication in DC‐SIGN expressing cells. PLoS ONE 8: e56164. 23424649 10.1371/journal.pone.0056164 PMC3570528

[cmi12249-bib-0031] Iborra, S. , Izquierdo, H.M. , Matinez‐Lopez, M. , Blanco‐Menendez, N. , Reis e Sousa, C. , and Sancho, D. (2012) The DC receptor DNGR‐1 mediates cross‐priming of CTLs during vaccinia virus infection in mice. J Clin Invest 122: 1268–1243. 10.1172/JCI60660 PMC3336985 22505455

[cmi12249-bib-0032] Iliev, I.D. , Funari, V.A. , Taylor, K.D. , Nguyen, Q. , Reyes, C.N. , Strom, S.P. , *et al*. (2012) Interactions between commensal fungi and the C‐type lectin receptor Dectin‐1 influence colitis. Science 336: 1314–1317. 22674328 10.1126/science.1221789 PMC3432565

[cmi12249-bib-0033] Ishikawa, E. , Ishikawa, T. , Morita, Y.S. , Toyonaga, K. , Yamada, H. , Takeuchi, O. , *et al*. (2009) Direct recognition of the mycobacterial glycolipid trehalose dimycolate, by C‐type lectin Mincle. J Exp Med 206: 2879–2888. 20008526 10.1084/jem.20091750 PMC2806462

[cmi12249-bib-0034] Ishikawa, T. , Itoh, F. , Yoshida, S. , Saijo, S. , Matsuzawa, T. , Gonoi, T. , *et al*. (2013) Identification of distinct ligands for the C‐type lectin receptors mincle and Dectin‐2 in the pathogenic fungus Malassezia. Cell Host Microbe 13: 477–488. 23601109 10.1016/j.chom.2013.03.008

[cmi12249-bib-0035] Kang, P.B. , Azad, A.K. , Torrelles, J.B. , Kaufman, T.M. , Beharka, A. , *et al*. (2005) The human macrophage MR directs MTB lipoarabinomannan‐mediated phagosome biogenesis. J Exp Med 202: 987–999. 16203868 10.1084/jem.20051239 PMC2213176

[cmi12249-bib-0036] Kerrigan, A.M. , and Brown, G.D. (2011) Syk‐coupled C‐type lectins in immunity. Trends Immunol 32: 151–156. 21334257 10.1016/j.it.2011.01.002 PMC3074083

[cmi12249-bib-0037] Konstantinov, S.R. , Smidt, H. , de Vos, W.M. , Bruijns, S.C. , Singh, S.K. , *et al*. (2008) S layer protein A of *Lactobacillus acidophilus* NCFM regulates immature dendritic cell and T cell functions. Proc Natl Acad Sci USA 105: 19474–19479. 19047644 10.1073/pnas.0810305105 PMC2592362

[cmi12249-bib-0038] Lähteenmäki, K. , Virkola, R. , Sarén, A. , Emödy, L. , and Korhonen, T.K. (1998) Expression of plasminogen activator pla of *Yersinia pestis* enhances bacterial attachment to the mammalian extracellular matrix. Infect Immun 66: 5755–5762. 9826351 10.1128/iai.66.12.5755-5762.1998 PMC108727

[cmi12249-bib-0039] Lee, W.B. , Kang, J.S. , Yan, J.J. , Lee, M.S. , Jeon, B.Y. , Cho, S.N. , and Kim, Y.J. (2012) Neutrophils promote mycobacterial trehalose dimycolate‐induced lung inflammation via the Mincle pathway. PLoS Pathog 8: e1002614. 22496642 10.1371/journal.ppat.1002614 PMC3320589

[cmi12249-bib-0040] Lefevre, L. , Lugo‐Villarino, G. , Meunier, E. , Valentin, A. , Olagnier, D. , Authier, H. , *et al*. (2013) The C‐type lectin receptors Dectin‐1, MR, and SIGNR3 contribute both positively and negatively to the macrophage response to *Leishmania infantum* . Immunity 38: 1–12. 23684988 10.1016/j.immuni.2013.04.010

[cmi12249-bib-0041] Lobato‐Pascual, A. , Saether, P.C. , Fossum, S. , Dissen, E. , and Daws, M.R. (2013) Mincle, the receptor for mycobacterial cord factor, forms a functional receptor complex with MCL and FcεRI‐γ. Eur J Immunol 43: 3167–3174. 23921530 10.1002/eji.201343752

[cmi12249-bib-0042] Long, K.M. , Whitmore, A.C. , Ferris, M.T. , Sempowski, G.D. , McGee, C. , Trollinger, B. , *et al*. (2013) Dendritic cell immunoreceptor regulates Chikungunya virus pathogenesis in mice. J Virol 87: 5697–5706. 23487448 10.1128/JVI.01611-12 PMC3648201

[cmi12249-bib-0043] McGuinness, D.H. , Dehal, P.K. , and Pleass, R.J. (2003) Pattern recognition molecules and innate immunity to parasites. Trends Parasitol 19: 312–319. 12855382 10.1016/s1471-4922(03)00123-5

[cmi12249-bib-0044] Maglinao, M. , Klopfleisch, R. , Seeberger, P.H. , and Lepenies, B. (2013) The C‐type lectin receptor DCIR is crucial for the development of experimental cerebral malaria. J Immunol 191: 2551–2559. 23918990 10.4049/jimmunol.1203451

[cmi12249-bib-0045] Marakalala, M.J. , Graham, L.M. , and Brown, G.D. (2010) The role of Syk/CARD9‐coupled C type lectin receptors in immunity to M. tuberculosis infections. Clin Dev Immunol 2010: 567571. 21274433 10.1155/2010/567571 PMC3025359

[cmi12249-bib-0046] Marakalala, M.J. , Vautier, S. , Potrykus, J. , Walker, L.A. , Shepardson, K.M. , Hopke, A. , *et al*. (2013) Differential adaptation of *C. albicans* in vivo modulates immune recognition by Dectin‐1. PLoS Pathog 9: e1003315. 23637604 10.1371/journal.ppat.1003315 PMC3630191

[cmi12249-bib-0047] Meevissen, M.H.J. , Yazdanbakhsh, M. , and Hokke, C.H. (2012) *Schistosoma mansoni* egg glycoproteins and C‐type lectins of host immune cells: Molecular partners that shape immune responses. Exp Parasitol 132: 14–21. 21616068 10.1016/j.exppara.2011.05.005

[cmi12249-bib-0048] Mesman, A.W. , de Vries, R.D. , McQuaid, S. , Duprex, W.P. , de Swart, R.L. , and Geijtenbeek, T.B.H. (2012) A prominent role for DC‐SIGN+ dendritic cells in initiation and dissemination of measles virus infection in non‐human primates. PLoS ONE 7: e49573. 23227146 10.1371/journal.pone.0049573 PMC3515571

[cmi12249-bib-0049] Meyer, S. , van Liempt, E. , Imberty, A. , van Kooyk, Y. , Geyer, H. , Geyer, R. , and Van Die, I. (2005) DC‐SIGN mediates binding of dendritic cells to authentic pseudo‐Lewis^Y^ glycolipids of *Schistosoma mansoni cercariae*, the first parasite‐specific ligand of DC‐SIGN. J Biol Chem 280: 37349–37359. 16155001 10.1074/jbc.M507100200

[cmi12249-bib-0050] Mishra, P.K. , Morris, E.G. , Garcia, J.A. , Cardona, A.E. , and Teale, J.M. (2013) Increased accumulation of regulatory granulocytic myeloid cells in mannose receptor C type 1‐deficient mice correlates with protection in a mouse model of neurocysticercosis. Infect Immun 81: 1052–1063. 23319563 10.1128/IAI.01176-12 PMC3639615

[cmi12249-bib-0051] Miyake, Y. , Toyonaga, K. , Mori, D. , Kakuta, S. , Hoshino, Y. , Oyamada, A. , *et al*. (2013) C‐type lectin MCL is an FcRgamma‐coupled receptor that mediates the adjuvanticity of mycobacterial cord factor. Immunity 38: 1050–1062. 23602766 10.1016/j.immuni.2013.03.010

[cmi12249-bib-0052] Netea, M.G. (2013) Training innate immunity: the changing concept of immunological memory in innate host defence. Eur J Clin Invest 43: 881–884. 23869409 10.1111/eci.12132

[cmi12249-bib-0053] Netea, M.G. , Quintin, J. , and van der Meer, J.W.M. (2011) Trained immunity: a memory for innate host defense. Cell Host Microbe 19: 355–361. 10.1016/j.chom.2011.04.006 21575907

[cmi12249-bib-0055] Piva, L. , Tetlak, P. , Claser, C. , Karjalainen, K. , Renia, L. , and Ruedl, C. (2012) Cutting edge: Clec9A+ dendritic cells mediate the development of experimental cerebral malaria. J Immunol 189: 1128–1132. 22732587 10.4049/jimmunol.1201171

[cmi12249-bib-0056] Powlesland, A.S. , Ward, E.M. , Sadhu, S.K. , Guo, Y. , Taylor, M.E. , and Drickamer, K. (2006) Widely divergent biochemical properties of the complete set of mouse DC‐SIGN‐related proteins. J Biol Chem 281: 20440–20449. 16682406 10.1074/jbc.M601925200

[cmi12249-bib-0057] Prasanphanich, N.S. , Mickum, M.L. , Heimburg‐Molinaro, J. , and Cummings, R.D. (2013) Glycoconjugates in host‐helminth interactions. Front Immunol 4: 240. 24009607 10.3389/fimmu.2013.00240 PMC3755266

[cmi12249-bib-0058] Quintin, J. , Saeed, S. , Martens, J.H.A. , Giamarellos‐Bourboulis, E.J. , Ifrim, D.C. , *et al*. (2012) *C. albicans* infection affords protection against reinfection via functional reprogramming of monocytes. Cell Host Microbe 12: 223–232. 22901542 10.1016/j.chom.2012.06.006 PMC3864037

[cmi12249-bib-0059] Redelinghuys, P. , and Brown, G.D. (2011) Inhibitory C‐type lectin receptors in myeloid cells. Immunol Lett 136: 1–12. 20934454 10.1016/j.imlet.2010.10.005 PMC3061320

[cmi12249-bib-0060] Ritter, M. , Gross, O. , Kays, S. , Ruland, J. , Nimmerjahn, F. , Saijo, S. , *et al*. (2010) *Schistosoma mansoni* triggers Dectin‐2, which activates the Nlrp3 inflammasome and alters adaptive immune responses. Proc Natl Acad Sci USA 107: 20459–20464. 21059925 10.1073/pnas.1010337107 PMC2996650

[cmi12249-bib-0061] Rogers, N.C. , Slack, E.C. , Edwards, A.D. , Nolte, M.A. , Schulz, O. , *et al*. (2005) Syk‐dependent cytokine induction by Dectin‐1 reveals a novel pattern recognition pathway for C Type lectins. Immunity 22: 507–517. 15845454 10.1016/j.immuni.2005.03.004

[cmi12249-bib-0062] Rothfuchs, A.G. , Bafica, A. , Feng, C.G. , Egen, J.G. , Williams, D.L. , Brown, G.D. , and Sher, A. (2007) Dectin‐1 interaction with MTB leads to enhanced IL‐12p40 production by splenic DCs. J Immunol 15: 3463–3471. 10.4049/jimmunol.179.6.3463 17785780

[cmi12249-bib-0063] Salazar, F. , Sewell, H.F. , Shakib, F. , and Ghaemmaghami, A.M. (2013) The role of lectins in allergic sensitization and allergic disease. J Allergy Clin Immunol 132: 27–36. 23534971 10.1016/j.jaci.2013.02.001

[cmi12249-bib-0064] Sancho, D. , and Reis e Sousa, C. (2012) Signaling by myeloid C‐type lectin receptors in immunity and homeostasis. Annu Rev Immunol 30: 491–529. 22224766 10.1146/annurev-immunol-031210-101352 PMC4480235

[cmi12249-bib-0065] Schlesinger, L.S. , Hull, S.R. , and Kaufman, T.M. (1994) Binding of the terminal mannosyl units of lipoarabinomannan from a virulent strain of M. tuberculosis to human macrophages. J Immunol 152: 4070–4079. 8144972

[cmi12249-bib-0066] Smits, H.H. , Engering, A. , van der Kleij, D. , de Jong, E.C. , Schipper, K. , *et al*. (2005) Selective probiotic bacteria induce IL‐10‐producing regulatory T cells in vitro by modulation dendritic cell function through DC‐SIGN. J Allergy Clin Immunol 115: 1260–1267. 15940144 10.1016/j.jaci.2005.03.036

[cmi12249-bib-0010] Sousa Mda, G. , Reid, D.M. , Schweighoffer, E. , Tybulewicz, V. , Ruland, J. , Langhorne J. , *et al*. (2011) Restoration of pattern recognition receptor co‐stimulation to treat chromoblastomycosis, a chronic fungal infection of the skin. Cell Host Microbe 9: 436–443. 21575914 10.1016/j.chom.2011.04.005 PMC3098964

[cmi12249-bib-0067] Steichen, A.L. , Binstock, B.J. , Mishra, B.B. , and Sharma, J. (2013) C‐type lectin receptor CLEC4D plays a protective role in resolution of Gram‐negative pneumonia. J Leukoc Biol 94: 393–398. 23709686 10.1189/jlb.1212622 PMC3747124

[cmi12249-bib-0068] Strasser, D. , Neumann, K. , Bergmann, H. , Marakalala, M.J. , Guler, R. , *et al*. (2012) Syk Kinase‐coupled C‐type lectin receptors engage protein kinase C‐δ to elicit Card‐9 adaptor‐mediated innate immunity. Immunity 36: 32–42. 22265677 10.1016/j.immuni.2011.11.015 PMC3477316

[cmi12249-bib-0069] Tanne, A. , Ma, B. , Boudou, F. , Tailleux, L. , Botella, H. , Badell, E. , *et al*. (2009) A murine DC‐SIGN homologue contributes to early host defense against M. tuberculosis. J Exp Med 206: 2205–2220. 19770268 10.1084/jem.20090188 PMC2757888

[cmi12249-bib-0070] Tserel, L. , Runnel, T. , Kisand, K. , Pihlap, M. , Bakhoff, L. , and Kolde, R. (2011) MicroRNA expression profiles of human blood monocyte‐derived dendritic cells and macrophages reveal miR‐511 as a putative positive regulator of Toll‐like receptor 4. J Biol Chem 286: 26487–26495. 21646346 10.1074/jbc.M110.213561 PMC3143613

[cmi12249-bib-0071] Tundup, S. , Srivastava, L. , and Harn, D.A. (2012) Polarization of host immune responses by helminth‐expressed glycans. Ann N Y Acad Sci 1253: E1–E13. 22974465 10.1111/j.1749-6632.2012.06618.x

[cmi12249-bib-0072] Van Vliet, S.J. , van Liempt, E. , Saeland, E. , Aarnoudse, C.A. , Appelmelk, B. , and Irimura, T. (2005) Carbohydrate profiling reveals a distinctive role for the C‐type lectin MGL in the recognition of helminth parasites and tumor antigens by dendritic cells. Int Immunol 17: 61–669. 10.1093/intimm/dxh246 15802303

[cmi12249-bib-9001] Yamasaki, S. , Ishikawa, E. , Sakuma, M. , Hara, H. , Ogata, K. , and Saito, T. (2008) Mincle is an ITAM‐coupled activating receptor that senses damaged cells. Nat Immunol 9: 1179–1188. 18776906 10.1038/ni.1651

[cmi12249-bib-0074] Zelenay, S. , Keller, A.M. , Whitney, P.G. , Schrami, B.U. , Deddouche, S. , *et al*. (2012) The dendritic cell receptor DNGR‐1 controls endolytic handling of necrotic cell antigens to favor cross‐priming of CTLs in virus‐infected mice. J Clin Invest 122: 1615–1627. 22505458 10.1172/JCI60644 PMC3336984

[cmi12249-bib-0075] Zelensky, A.N. , and Gready, J.E. (2005) The C‐type lectin‐like domain superfamily. FEBS J 272: 6179–6217. 16336259 10.1111/j.1742-4658.2005.05031.x

[cmi12249-bib-0076] Zhang, S.S. , Park, C.G. , Zhang, P. , Bartra, S.S. , Plano, G.V. , *et al*. (2008) Plasminogen activator PLA of *Yersinia pestis* utilizes murine DEC‐205 as a receptor to promote dissemination. J Biol Chem 283: 31511–31521. 18650418 10.1074/jbc.M804646200 PMC2581554

